# Supplementary material for: Rare Prenyllipids in Wild St. John’s Wort During Three Harvest Seasons
Source: Molecules. 2025 Feb 15;30(4):901. doi: 10.3390/molecules30040901 (PMC11858351; doi:10.3390/molecules30040901)
Supplement: Supplementary file 1 [file molecules-30-00901-s001.zip › molecules-3436121-supplementary.pdf]

Figure S1. Wild *Hypericum perforatum* L. aerial parts.

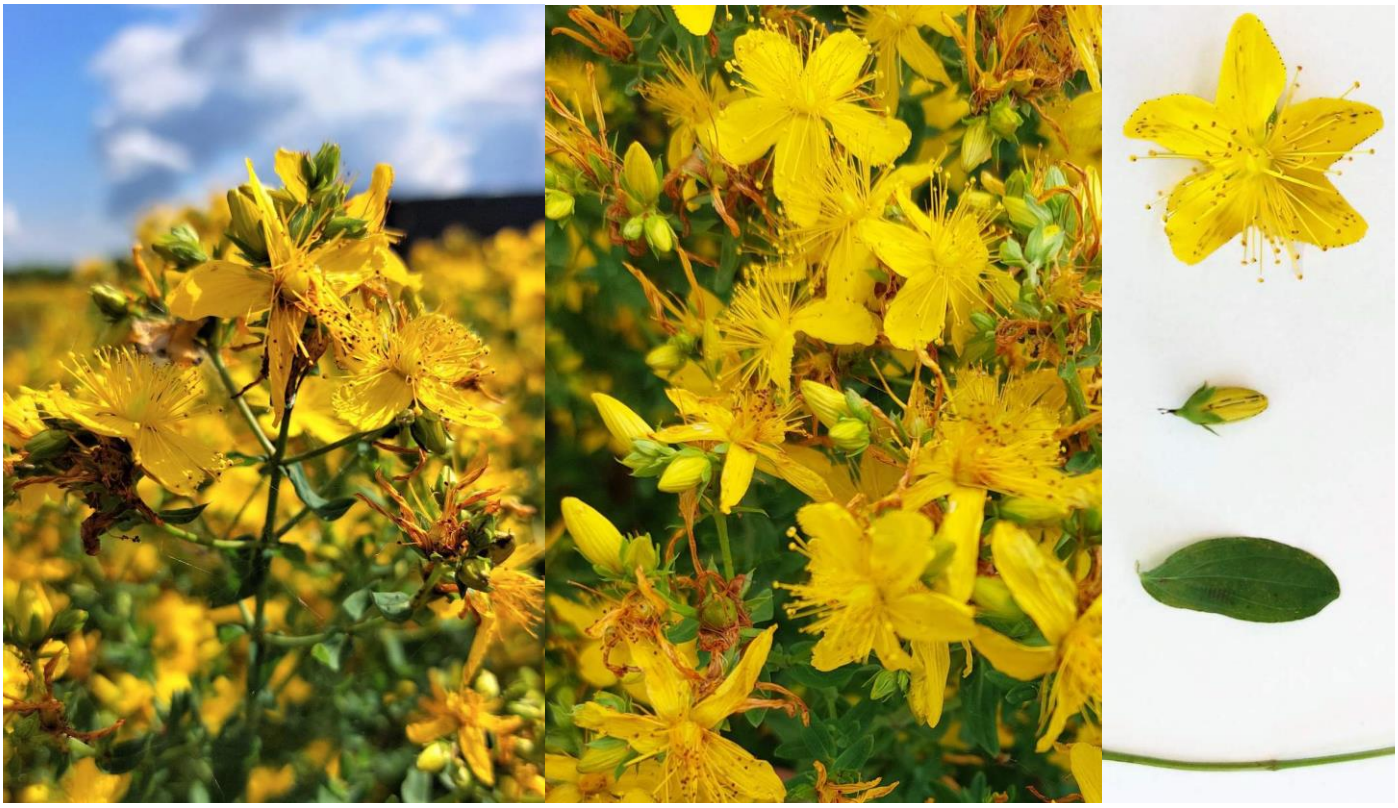

Figure S2. Chromatograms of the tocotrienol (T3) and tocopherol (T) homologues' (α, β, γ, and δ) separation by RP-HPLC/FLD in wild *H. perforatum* stems, leaves, flower buds, flowers and standards.

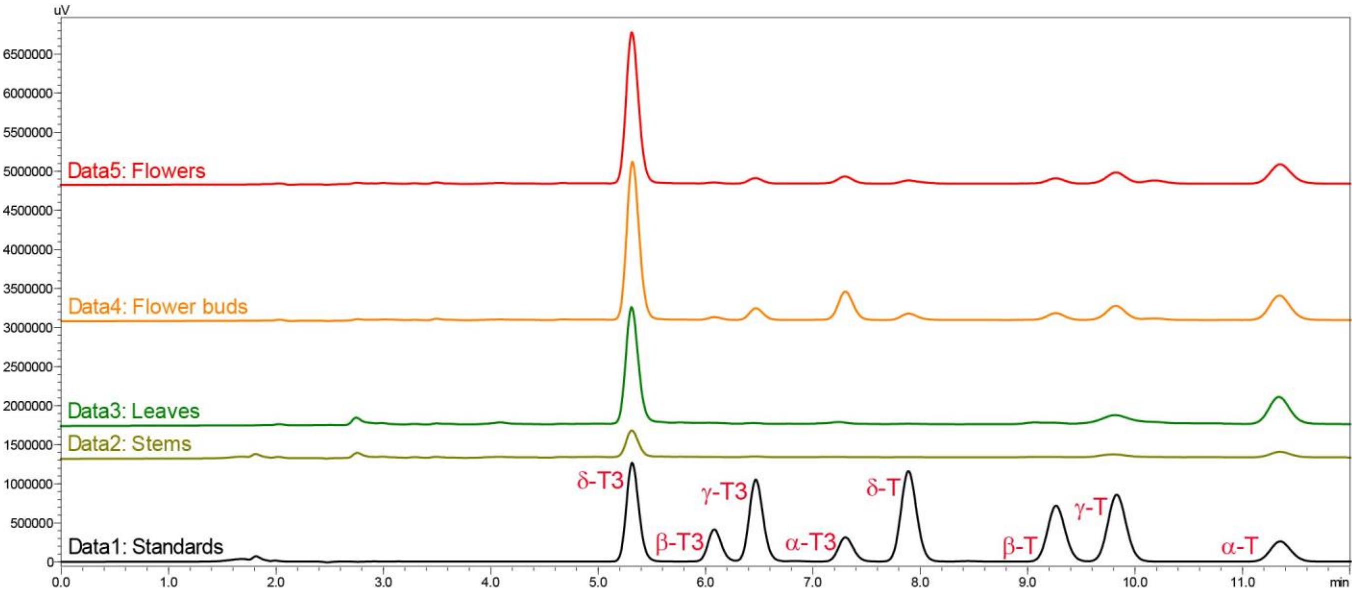

Table S1. Contents and ratios of tocopherols and tocotrienols in leaves, flower buds, and flowers of wild *H. perforatum* harvested during 2022–2024.

Plant part	Tocochromanols, mg/100 g dw											Ratio Ts/T3s
	α-T	β-T	γ-T	δ-T	α-T3	β-T3	γ-T3	δ-T3	Total Ts	Total T3s	Total Ts+T3s	
**Leaves**												
Min	38.8	0.1	0.8	nd	nd	nd	nd	8.3	41.6	9.9	52.4	1.8
Max	93.1	1.0	4.4	1.1	1.9	0.4	1.5	22.7	96.7	23.6	111.8	8.0
Average	61.3	0.4	2.0	0.1	0.7	nd	0.3	14.5	63.8	15.5	79.4	4.3
STDEV	16.0	0.2	0.9	0.2	0.5	0.1	0.3	3.6	16.2	3.5	15.6	1.5
Coefficient of variation	26.1	46.9	44.0	153.3	78.0	177.4	86.4	24.7	25.3	22.3	19.7	35.5
**Flower buds**												
Min	22.3	1.7	3.1	0.5	11.8	nd	0.3	28.9	30.6	45.1	83.3	0.5
Max	38.0	6.4	11.7	5.0	32.6	3.0	7.3	47.1	53.6	81.6	123.8	0.9
Average	31.8	3.8	5.6	1.7	23.7	1.0	3.4	34.8	42.9	62.9	105.8	0.7
STDEV	3.4	1.0	1.8	0.8	5.3	0.7	2.0	4.0	4.3	8.3	10.0	0.1
Coefficient of variation	10.6	25.8	32.7	47.4	22.1	67.9	58.4	11.5	10.0	13.1	9.5	14.9
**Flowers**												
Min	21.1	0.7	2.3	0.1	0.9	nd	0.1	26.7	26.5	28.4	60.2	0.7
Max	33.3	5.6	8.9	4.1	7.5	1.4	2.2	41.7	49.6	48.1	87.8	1.4
Average	27.9	3.4	5.2	1.5	3.6	0.2	0.8	30.8	38.1	35.5	73.5	1.1
STDEV	3.0	1.0	1.5	0.7	1.6	0.3	0.5	3.5	4.7	4.2	6.5	0.2
Coefficient of variation	10.6	31.1	29.4	45.2	44.7	137.4	65.1	11.5	12.4	11.8	8.9	15.3

Average values and standard deviations correspond to nine biological samples of each aerial part of *H. perforatum* (n = 9). T, tocopherol; T3, tocotrienol; tr, trace amount (below 0.05 mg/100 g dw); dw, dry weight.

Table S2. Two-factorial analysis of variance for tocochromanol contents in three aerial parts (leaves, flower buds, and flowers) of wild *H. perforatum* harvested during 2022–2024.

Effect	Sum of squares (SS)	*df*	Mean of square (MS)	*F* statistic	*p* value
**α-T**					
Year	890	2	455	6	0.002253
Plant part	35 923	2	17 962	256	<0.000001
Plant part × Year	2 947	4	734	11	<0.000001
Error	10 734	153	70		
**β-T**					
Year	6	2	3	4	0.015737
Plant part	366	2	183	278	<0.000001
Plant part × Year	4	4	1	2	0.203635
Error	101	153	0.7		
**γ-T**					
Year	36	2	18	9	0.000162
Plant part	430	2	215	111	<0.000000
Plant part × Year	15	4	4	2	0.101658
Error	295	153	2		
**δ-T**					
Year	4	2	2	5	0.009365
Plant part	77	2	38	104	<0.000001
Plant part × Year	1	4	0.3	0.8	0.515486
Error	57	153	0.4		
**α-T3**					
Year	35	2	18	2	0.164444
Plant part	17 015	2	8 507	882	<0.000001
Plant part × Year	108	4	27	3	0.028327
Error	1 475	153	10		
**β-T3**					
Year	0.2	2	0.1	0.7	0.491939
Plant part	27	2	13	79	<0.000001
Plant part × Year	0.9	4	0.2	1	0.238323
Error	26	153	0.2		
**γ-T3**					
Year	8	2	4	3	0.050136
Plant part	292	2	146	109	<0.000001
Plant part × Year	12	4	3	2	0.075700
Error	205	153	1		
**δ-T3**					
Year	6	2	3	0.2	0.801279
Plant part	12 543	2	6 271	474	<0.000001
Plant part × Year	170	4	42	3	0.014459
Error	2 023	153	13		
